# Long-Term Stability and Histologic Evaluation of Orthodontically Driven Osteogenesis (ODO): A Preliminary Retrospective Study

**DOI:** 10.3390/jcm14196896

**Published:** 2025-09-29

**Authors:** Federico Brugnami, Simonetta Meuli, Valentina Ventura, Davide Gentile

**Affiliations:** 1 Private Practice, 00195 Rome, Italy; 2 Postgraduate School of Orthodontics, Catholic University of the Sacred Heart, Largo Agostino Gemelli 8, 00168 Rome, Italy; 3 Department of Chemical Science and Technologies, Materials for Sustainable Development—Dentistry, University of Rome Tor Vergata, 00133 Rome, Italy

**Keywords:** periodontology, bone augmentation, clear aligners, corticotomy, histology, orthodontically driven osteogenesis, orthodontics, periodontally accelerated osteogenic orthodontics

## Abstract

**Background**: Orthodontically driven osteogenesis (ODO) is a surgical tunnel modification of periodontally accelerated osteogenic orthodontics (PAOO), combining selective corticotomy with bone grafting in sequential and/or segmental fashion. This is a minimally invasive approach that enhances periodontal health and allows orthodontic tooth movement beyond the original alveolar envelope. Considering the lack of long-term three-dimensional data on orthodontically driven osteogenesis (ODO), this study aims to quantitatively assess the long-term stability of alveolar bone and buccal cortical thickness following ODO, using CBCT imaging. The null hypothesis is that ODO does not result in significant changes in alveolar bone volume or cortical thickness over a seven-year follow-up period. **Methods:** Twenty patients (13 females, 7 males; mean age 27.4 ± 5.3 years) who had undergone orthodontically driven osteogenesis (ODO) using a minimally invasive tunnel approach and segmental corticotomy protocol followed by clear aligner therapy were retrospectively evaluated. The mean follow-up period after treatment was 7 years (range: 5–15 years). Cone beam computed tomography (CBCT) scans were obtained at one year postoperatively (T1) and again at the long-term follow-up visit (T2). Buccal bone thickness measurements were taken at standardized levels (3 mm, 5 mm, and 7 mm apical to the cementoenamel junction) and compared between T1 and T2 to evaluate bone stability over time. In addition, histologic evaluation of the previously grafted area was performed in two patients: one sample was collected during an alveolar ridge augmentation procedure six months after ODO, and the other during orthognathic surgery eight months after ODO. The samples were analyzed to assess new bone formation and integration of graft material. **Results**: Radiographic analysis showed long term stability of the new bone support. Histologic examination showed newly formed lamellar and reticular bone. Bone marrow showed no inflammatory infiltration, and bone particles were still detectable but incorporated in the newly created bone. **Conclusions**: Based on these findings, ODO appears to be a promising technique that could induce stable bone osteogenesis. A larger cohort study can enhance the evidence of these promising results to popularize this technique.

## 1. Introduction

### 1.1. Periodontally Accelerated Osteogenic Orthodontics

Bone engineering has become a cornerstone of modern orthodontics, particularly in overcoming the anatomical limitations of the alveolar housing. Such limitations can restrict the extent and effectiveness of orthodontic treatment, especially in cases involving severe malocclusions or compromised periodontal conditions [1]. Among the available approaches, periodontally accelerated osteogenic orthodontics (PAOO) has been widely studied for its ability to enhance orthodontic tooth movement while preserving periodontal health. PAOO combines full-thickness flap reflection, selective corticotomy, bone grafting, and orthodontic force application to stimulate a regional acceleratory phenomenon (RAP) and accelerate bone remodeling [2].

### 1.2. Orthodontically Driven Osteogenesis

Orthodontically driven osteogenesis (ODO) represents a modification of the PAOO protocol. Unlike conventional PAOO, which requires extensive flap reflection, ODO employs a minimally invasive tunnel approach, limiting soft tissue trauma and improving patient comfort. In addition, ODO is carried out in a sequential or segmental fashion, focusing only on the areas where teeth are planned to move beyond the native bony envelope. This targeted method minimizes surgical morbidity while still inducing the desired biological response and allowing precise bone augmentation. The primary objective of ODO, like PAOO, is to expand and remodel the alveolar housing, thereby reinforcing skeletal support around the teeth and increasing available bone volume. The surgical phase induces transient osteopenia and elicits a localized inflammatory response, triggering RAP. These biological events accelerate bone turnover, enhance the rate of tooth movement, and reduce overall treatment duration [3]. Importantly, this modified approach not only shortens treatment time but also mitigates adverse periodontal effects, including marginal bone loss, root resorption, and gingival recession—complications often seen during alignment of severely crowded teeth or during decompensation and retraction phases [4]. ODO may therefore be particularly advantageous for patients with a compromised periodontium, where conventional orthodontic mechanics could worsen periodontal status.

### 1.3. Rationale and Study Objectives

While several studies have investigated PAOO outcomes using two-dimensional imaging (e.g., periapical radiographs), these modalities offer limited insight into three-dimensional bone changes [5,6,7]. Cone beam computed tomography (CBCT) enables more accurate assessment of alveolar bone morphology and density. However, few studies have applied CBCT specifically to this envelope technique and even fewer have included long-term follow-up to evaluate the stability of outcomes. Wang et al. used CBCT to evaluate the periodontal preservation achieved by PAOO after presurgical multibracket orthodontics [8]. Considering the lack of long-term three-dimensional data on ODO, this study aims to quantitatively assess the long-term stability of alveolar buccal bone thickness following ODO using CBCT imaging. The null hypothesis is that ODO does not result in significant changes in alveolar bone volume or cortical thickness over a seven-year follow-up period.

## 2. Materials and Methods

### 2.1. Study Design

This study was considered exempt from institutional review board (IRB) oversight in accordance with current regulations for research conducted in a private practice setting in Italy. A formal sample size calculation was performed for the primary outcome, buccal cortical thickness. Assuming a clinically relevant difference of 0.3 mm between T1 and T2, a standard deviation of 0.5 mm, a significance level of 0.05, and a power of 80%, the required sample size was estimated to be approximately 22 patients. Thus, the present sample of 20 subjects, who underwent ODO from January 2012 to January 2015, was considered adequate for the exploratory evaluation of long-term alveolar changes after this surgical procedure [9]. Twenty patients (13 females and 7 males; mean age 27.4 ± 5.3 years), were retrospectively evaluated at baseline and five years after undergoing the ODO procedure. Patients were eligible for inclusion if they required orthodontic treatment with clear aligners to address dental crowding, presented with a compromised periodontal status, and had full dentition without missing teeth or clinical mobility. Exclusion criteria included patients undergoing pharmacological therapies that could affect bone metabolism or periodontal conditions, as well as any systemic or local factors potentially influencing bone remodeling or healing capacity. Cone beam computed tomography (CBCT) scans were performed one year after the ODO procedure (T1) and again at the seven-year follow-up (T2). In two of these patients, bone samples were collected for histological evaluation. The sampling was performed based on the availability of a follow-up CBCT.

### 2.2. Surgical Protocol

The surgical procedure was carried out by a single, experienced clinician (F.B.) following a well-established tunnel regenerative approach designed to minimize trauma and enhance healing. Initially, a precise vertical incision was made at the mucogingival junction, ensuring that the surgical site could be adequately exposed while minimizing disruption to the surrounding soft tissues. The incision was strategically planned to avoid damaging the gingival papillae, which are critical for maintaining periodontal aesthetics and function. Following the initial incision, a tunnel elevation technique was employed to lift the soft tissue flap, providing sufficient access to the underlying alveolar bone without the need for large flap reflections, thereby preserving the integrity of the gingival tissue. Once the flap was elevated, a segmental corticotomy was performed in the region of the alveolar bone that was to be subjected to orthodontic movement. This step was crucial to enhance the regional acceleratory phenomenon (RAP) and promote the desired bone remodeling. A #15 Bard–Parker surgical blade was used to make sulcular incisions along the planned treatment site, ensuring precision and minimal soft tissue injury. The sulcular incisions were carefully designed to preserve the surrounding gingival papillae, maintaining both functional and esthetic outcomes post-surgery. The corticotomy was then carried out using a piezoelectric scalpel, which allows for controlled and minimally invasive bone cuts. The piezoelectric scalpel’s ultrasonic vibration provides superior precision compared to traditional methods, minimizing damage to adjacent tissues such as the periodontal ligament and surrounding soft tissues and reducing the risk of thermal injury to the bone. Following this procedure, a dermal substitute specifically designed to aid in tissue regeneration and promote healing was placed at the gingival margin. This dermal substitute was securely fixed in position using continuous suspension vertical mattress sutures, which provided both stability and optimal tension to support the healing process. The dermal substitute not only protected the grafting material but also enhanced the soft tissue healing and re-epithelialization in the surgical area. Bone grafting was performed using 0.5 cc of xenogeneic bovine bone graft material, chosen for its osteoconductive properties and ability to integrate with the existing bone to promote new bone formation. The graft material was carefully placed beneath the dermal substitute to augment the alveolar bone and support the newly formed bone structure as it remodeled under the influence of the applied orthodontic forces. This step was essential for enhancing the volume and density of the alveolar bone, providing a more stable foundation for orthodontic tooth movement. Finally, the vertical incisions were meticulously reapproximated using 5-0 Vicryl sutures, which are absorbable and offer excellent tensile strength, ensuring proper closure and minimizing the risk of wound dehiscence. These sutures helped achieve precise tissue alignment, promoting optimal healing of both the soft and hard tissues. The entire procedure was conducted under sterile conditions, ensuring minimal risk of infection and promoting a smooth recovery phase for the patient [7].

### 2.3. Orthodontic Protocol

The orthodontic therapy consisted of the alignment and coordination of the dental arches through a clear aligner system. All treatments were carried out by the same clinician (S.M.) to ensure standardization of the protocol and reduce operator variability. The therapeutic sequence was structured as follows:

1. Initial assessment and diagnostic workflow. Following a thorough clinical evaluation, comprehensive diagnostic records were obtained. These included digital scans of the dental arches, standardized intraoral and extraoral photographs, and radiographic documentation consisting of both panoramic (orthopantomography) and lateral cephalometric radiographs. Based on these data, a detailed analysis of the malocclusion was performed, and a virtual three-dimensional treatment simulation was generated using the ClinCheck^®^ Pro 6.0 software (Align Technology, San Jose, CA, USA). This digital setup allowed for precise visualization of tooth movement, prediction of occlusal outcomes, and assessment of arch form changes prior to the surgical phase.
2. Treatment planning with the Invisalign^®^ Comprehensive package—The “Comprehensive” aligner system (Align Technology, San Jose, CA, USA) was selected, as it provides flexibility for multi-phase treatments and the possibility of refinements when required. The planned orthodontic movements included dental arch expansion and coordination aimed at resolving crowding and achieving proper arch form alignment. The treatment protocol deliberately excluded interproximal enamel reduction (IPR) and tooth extractions, thereby favoring a non-invasive and expansion-based strategy consistent with the regenerative potential induced by ODO.
3. Aligner wear protocol and compliance instructions—Patients were instructed to wear their aligners for 20–22 h per day, removing them only during meals and oral hygiene procedures. Aligner changes were scheduled on a 7-day basis to ensure a continuous and controlled force application, harmonized with the accelerated bone remodeling process induced by the surgical procedure. On average, each patient required approximately 30 aligners per arch to complete the active orthodontic phase. Patient adherence to the prescribed wear time was emphasized as a critical factor for the success of treatment, especially in the context of surgically accelerated orthodontic tooth movement.
4. Retention strategy—Following completion of the active orthodontic therapy, a retention phase was implemented to maintain the achieved results and prevent relapse. Each patient was provided with a vacuum-formed retainer (VFR) tailored to their post-treatment dental arches. Patients were instructed to wear the retainers nightly on a long-term basis. This retention approach was chosen for its effectiveness, comfort, and minimal impact on oral hygiene, ensuring stability of the orthodontic results obtained after ODO.

### 2.4. Radiological Evaluation

**Figure 1:**
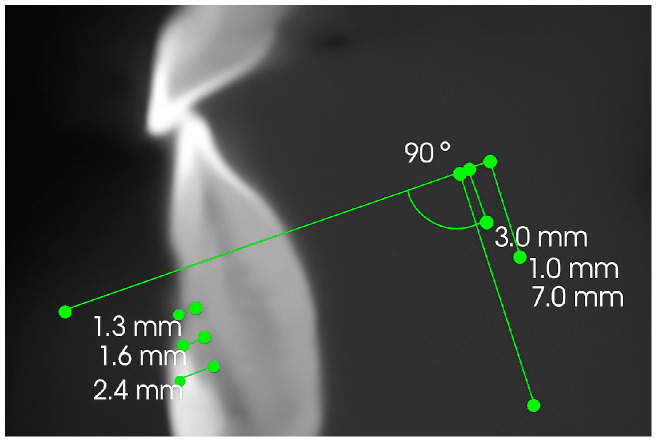
Measurements of marginal bone stability on CBCT.

Each investigation was performed using an 8200 3D CBCT (Carestream Health, Rochester, NY, USA) unit following the same parameters: the exposed volume was 50 mm by 30 mm, involving the dental region where ODO was completed. The measurements were taken using CS3D Imaging software 3.8.7. (Carestream Health, USA) to extract the image volume of perpendicular slices in axial, coronal, and sagittal planes. Following the protocol proposed by Lund et al., reconstructions were set to have the axial slices perpendicular to the long axis [7]. In this way, the bone marginal level (BM) was visualized in relation to CEJ in each view. Measurements of thickness of the buccal plate were performed at 3 mm, 5 mm, and 7 mm at the CEJ of lower incisors and canines at T1 and T2, tracing a reference line between CEJ and buccal/lingual surface. Radiographic measurements were carried out by two examiners (F.B. and S.M.), who repeated the same assessments after a two-week interval. The intraclass correlation coefficient for inter-examiner agreement ranged between 0.81 and 0.92, demonstrating the reliability and reproducibility of the radiographic analysis. According to Dahlberg’s formula, measurement errors varied from 0.12 to 0.29 mm for linear values (Figure 1). The difference between T1 and T2 represents the stability of thickness during follow up. For the statistical analysis, Student’s *t* test for the difference of group means was performed, considering a *p* value of <0.05 and using Statistical Package for Social Sciences (SPSS) software version 21.0.

### 2.5. Histological Evaluation

Histological evaluation was performed in two patients who had previously undergone orthodontically driven osteogenesis (ODO). Written informed consent was obtained from both patients prior to sampling. In the first case, a bone specimen was harvested during an alveolar ridge augmentation procedure combined with implant placement, carried out six months after the completion of ODO. In the second case, the sample was collected during bilateral sagittal split osteotomy performed for the correction of a skeletal Class III malocclusion, eight months after ODO. In both instances, the biopsies were retrieved from clinically relevant surgical sites without additional patient morbidity, in accordance with ethical principles for human tissue research. The bone specimens were immediately fixed in 10% buffered formalin, decalcified, and processed for standard histological examination following established protocols [10]). Sections were stained with hematoxylin and eosin (H&E) to evaluate general tissue morphology, and additional staining techniques were employed, when necessary, to assess bone remodeling and the quality of newly formed tissue.

## 3. Results

### 3.1. Radiological Outcomes

A total of twenty patients (200 teeth) underwent orthodontic movement forward within their native bony envelope after ODO. The mean follow-up was 7 years. CBCT evaluation of buccal plate thickness showed minimal changes between the post-ODO baseline and the 7-year follow-up. At the 3 mm reference point, values decreased from 1.93 ± 0.55 mm to 1.91 ± 0.53 mm; at the 5 mm point, from 2.15 ± 0.62 mm to 2.14 ± 0.63 mm; and at the 7 mm point, from 3.19 ± 0.86 mm to 3.16 ± 0.86 mm. None of these differences reached statistical significance (*p* > 0.05). The complete data are summarized in Table 1. These findings indicate that alveolar bone volume and cortical thickness remained stable over a 7-year period, supporting the null hypothesis. However, the limited sample size may have reduced the statistical power of the analysis.

### 3.2. Histological Findings

**Figure 2:**
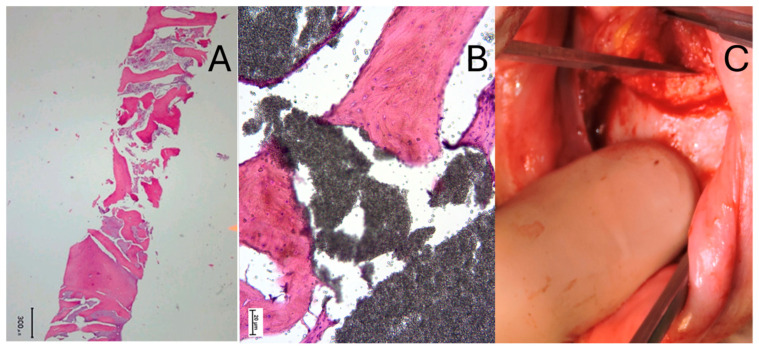
Histological examination of two bone samples from different patients. (**A**) Sample obtained during an implant placement (300 μm). (**B**) Sample obtained during orthognathic surgery. (**C**) Intraoperatory record [11].

Histological evaluation was performed in two patients after the patients’ permission and the extraction of a bone sample. The first one was taken during alveolar ridge augmentation and implant placement, six months after ODO (Figure 2A). The second one was performed during the bilateral sagittal osteotomy of a skeletal Class III malocclusion, eight months after ODO (Figure 2B). Histological analysis revealed the presence of viable bone, with remnants of the original graft material embedded within newly formed woven and lamellar bone. According to this histological evaluation, the inner portion of the graft turned into newly regenerated bone, with some portion of bone marrow. It could be likely that the slow resorption particles of the graft provided a filler to allow the long-term stability of the soft tissues. Moreover, during the maxillofacial procedure, the surgeon needed to use a mallet to drive the chisel, as the bone graft had fully integrated with the surrounding tissue (Figure 2C).

## 4. Discussion

### 4.1. Periodontal Effects of Orthodontic Treatment

The effects of orthodontic treatment on periodontal status remain controversial. Numerous studies have reported a higher incidence of gingival recession in patients undergoing dental arch expansion through orthodontic movements [11,12]. Buccal tipping or excessive proclination, particularly in the anterior region, has been associated with increased risks of soft tissue recession and bone dehiscence [13,14,15]. Alveolar bone resorption is more likely when teeth are moved beyond the alveolar housing, especially in thin biotypes or with limited buccal support, which can compromise both aesthetic and functional outcomes, particularly in adult patients [16,17]. Nevertheless, similar patterns of alveolar bone loss and gingival recession have also been observed in untreated patients with severe crowding (>5 mm), suggesting that malocclusion itself may predispose to unfavorable periodontal conditions [6,18]. This aspect highlights the multifactorial etiology of periodontal changes and the difficulty of isolating the specific contribution of orthodontic mechanics from patient-related risk factors such as bone morphology, gingival biotype, and hygiene [19].

### 4.2. Insights from Orthodontically Driven Osteogenesis

Recent studies on ODO have offered new perspectives on the role of surgical adjuncts in reducing the risks associated with orthodontic treatment in patients with periodontal involvement. Park et al. demonstrated that this procedure leads to long-term improvements in bone density and cortical thickness, suggesting that the procedure not only accelerates tooth movement but also enhances alveolar stability over time [20]. Similarly, Jiang et al., in a five-year follow-up study comparing ODO with conventional orthodontics, reported superior periodontal outcomes in the ODO group, including reduced incidence of gingival recession and greater post-treatment stability [21]. These findings support the hypothesis that biologically guided surgical facilitation may expand the limits of safe orthodontic tooth movement and improve long-term tissue health. Furthermore, the authors highlighted the benefits of combining ODO with temporary anchorage devices in cases requiring complex or en masse movements, suggesting that the synergistic effect of enhanced bone turnover and skeletal anchorage may broaden the scope of movements that can be predictably achieved without compromising periodontal support. Lee et al. also showed that ODO significantly shortens overall treatment duration while maintaining periodontal health, reinforcing its utility as a clinically efficient adjunct [4].

### 4.3. Limitations and Strengths

Despite these promising insights, the literature on ODO assessed through CBCT remains limited, and studies with long-term follow-up are still rare [22]. The histological evaluation in two patients provides a dual perspective: while the small sample size represents a clear limitation and prevents statistical analysis, it also constitutes a distinctive feature of our study, offering direct tissue-level evidence of new bone formation in augmented areas. Nevertheless, these findings should be interpreted with caution, as histological samples from only two individuals cannot be considered representative of the broader patient population. Another limitation is that the target sample size initially planned for the study was not reached, reducing the overall statistical power and potentially limiting the generalizability of our results. Moreover, CBCT analysis, although widely accepted in clinical and research settings, remains an indirect surrogate for histological validation and may not fully capture the biological complexity of bone remodeling processes. Finally, the absence of a control group and the exploratory design further emphasize that our results should be regarded as preliminary. CBCT imaging, however, did confirm volumetric stability over time, supporting the clinical relevance of these findings. To our knowledge, no comparable studies with extended follow-up exist, highlighting both the novelty and the pilot nature of our research. A key innovation of the present study is the integration of CBCT imaging with ClinCheck^®^ software, enabling three-dimensional evaluation of the relationship between dental roots and alveolar bone [23,24]. By overcoming the inherent limitations of two-dimensional radiographs, CBCT-based planning fosters a more biologically conscious and individualized approach to orthodontic care [25].

### 4.4. Future Directions and Clinical Implications

Future investigations should expand sample size, include multicentric randomized designs, and integrate advanced imaging technologies such as AI-driven volumetric segmentation to enhance measurement precision and provide deeper insights into bone remodeling dynamics. From a clinical perspective, the accumulating evidence—including the present study—suggests that ODO protocols may help expand the limits of safe orthodontic movement, reduce risks of gingival recession [26], and provide predictable alternatives for adult or periodontally vulnerable patients. Additionally, future studies should investigate patient-centered outcomes such as comfort, quality of life, treatment duration, and cost-effectiveness, which are essential for translating these protocols into routine orthodontic practice.

## 5. Conclusions

When alveolar boundaries are exceeded, ODO may represent a potential option. In this preliminary study, our data indicate that ODO can be associated with stable outcomes over an average follow-up of 7 years. Histological observations in a very limited number of patients suggest the possibility of new bone formation; however, these findings must be interpreted with caution.

Modern diagnostic tools, including three-dimensional imaging and digital planning, remain important to guide treatment and reduce potential risks. In selected cases where dental movements extend beyond anatomical limits, ODO may offer a feasible approach, but further research with larger cohorts and more extensive histological evaluation is necessary to confirm these results and better define the clinical indications of this technique.

## Figures and Tables

**Table 1:** Cone beam computed tomography (CBCT) scans were performed for measurements of thickness of buccal plates one year after the ODO procedure (T1) and again at the seven-year follow-up (T2) at different distances from CEJ on CBCT.

Distance from CEJ	3 mm		5 mm		7 mm	
Time	T1	T2	T1	T2	T1	T2
Measurements (mm)	1.93	1.91	2.15	2.14	3.19	3.16
Mean (mm)	0.55	0.53	0.62	0.63	0.86	0.86
(±Standard deviation)	0.88	0.88	0.95	0.95	0.89	0.89

Legend: ODO—orthodontically driven osteogenesis; CEJ—cement-enamel junction.

## Data Availability

Data and materials supporting the results or analyses presented in the present paper are available upon reasonable request to the corresponding author.
